# Secondary peripheral chondrosarcoma arising in solitary osteochondroma: variables influencing prognosis and survival

**DOI:** 10.1186/s13023-022-02210-2

**Published:** 2022-02-22

**Authors:** Alberto Righi, Marina Pacheco, Stefania Cocchi, Sofia Asioli, Marco Gambarotti, Davide Maria Donati, Andrea Evangelista, Maria Gnoli, Manuela Locatelli, Marina Mordenti, Manila Boarini, Evelise Brizola, Elena Pedrini, Luca Sangiorgi

**Affiliations:** 1 Department of Pathology, IRCCS Istituto Ortopedico Rizzoli, Bologna, Italy; 2 Department of Pathology, Complejo Hospitalario Metropolitano CSS, Panama, Panama; 3 Department of Biomedical and Neuromotor Sciences (DIBINEM), University of Bologna, Bologna, Italy; 4 Department of Orthopaedic Oncology, IRCCS Istituto Ortopedico Rizzoli, Bologna, Italy; 5 Unit of General Affairs, IRCCS Istituto Ortopedico Rizzoli, Bologna, Italy; 6 Department of Rare Skeletal Disorders, IRCCS Istituto Ortopedico Rizzoli, Via Pupilli 1, 40136 Bologna, Italy

**Keywords:** Secondary peripheral chondrosarcoma (SPC), Solitary osteochondroma, Prognostic factors

## Abstract

**Background:**

Secondary peripheral chondrosarcomas arising in solitary osteochondromas is an unusual complication, reported in small series. In this study, we aimed to present our experience with this rare variant of chondrosarcoma and compare results with already published data in order to determine prognostic factors for overall and disease-free survival.

**Methods:**

The case study includes retrospective data from patients diagnosed at a single institution from 1943 to 2019. Clinical data were collected reviewing all available medical records from first to last follow-up visits. To exclude the presence of the Multiple Osteochondroma Hereditary Syndrome, few patients, with a suspect of a familial form of the disease, were evaluated for the presence of germline heterozygous variants in *EXT1* and *EXT2* genes. Results were summarized using descriptive statistics and statistical analysis were performed to reveal associations between variables.

**Results:**

Two hundred and fourteen secondary peripheral chondrosarcomas that arose exclusively from solitary osteochondromas diagnosed in a multidisciplinary setting at the IRCCS Istituto Ortopedico Rizzoli were retrospectively identified, 66.4% males and 33.6% females with a median age at diagnosis of 38 years. The local recurrence rate was 17.3%, while the metastases one was 5.1%. Besides age, a high histologic grade is the only factor associated with worse 5-year and 10-year overall survival (log-rank *p* = 0.0005, HR = 3.74; 95% CI 1.69–8.26). Moreover, high histological grade (HR = 3.75; 95% CI = 1.69–8.34; *p* = 0.001) and surgical debulking (HR = 3.71; 95% CI = 1.57–8.79; *p* = 0.003) were associated with a significantly worse disease-free survival.

**Conclusions:**

Our study confirm the low-grade behavior of secondary peripheral chondrosarcomas and demonstrate that the best choice of treatment for those arising in solitary osteochondromas is the wide surgical excision, when possible. Location per se is not a factor that affects prognosis, while the accurate histological grade assessment is correlated with the tumor aggressiveness and a long term follow up is necessary for this rare variant of chondrosarcoma.

## Background

Secondary peripheral chondrosarcomas (SPCs) are malignant cartilaginous neoplasms that arise from the chondroid cap of pre-existent osteochondromas. Malignant transformation of solitary osteochondromas (SOC) is rare. Its incidence is not well established, ranging from about 1–7.6% in the published series [1–6], although most authors agree that patients with SOC have 1–2% risk of developing a secondary chondrosarcoma.

In the Rizzoli experience, secondary peripheral chondrosarcomas that originate from SOC represent approximately 13% of all chondrosarcomas seen in the Institute, occur preferentially in the third and fourth decades of life and exhibit a male preponderance, as it is well-established [2, 4–7]. Pain, progressive tumor growth after skeletal maturation, ill-defined and irregular margins, areas of radiolucency within an otherwise well-mineralized osteochondroma and cartilage cap thickness greater than 2 cm on imaging are signs of possible malignant transformation.

The most frequent sites of involvement of peripheral chondrosarcomas secondary to solitary osteochondromas are the ilium (20.5%), proximal and distal metaphysis of femur (19.6%), pubic bone (15.3%), proximal humerus (12%), proximal metaphysis of tibia and the scapula (6.8%, each) [4–6].

To date, the Tsuda et al. [7] publication of a series of 51 patients is the only available study that investigated the factors that affect the prognosis of secondary peripheral chondrosarcomas arising from pre-existing osteochondromas, either solitary or in the context of hereditary Multiple Osteochondromas (MO). Even though chondrosarcomatous transformation of a SOC is an unusual complication, the pre-existent benign condition is common enough to deserve special attention.

In this study, we aimed to present our experience with 214 secondary peripheral chondrosarcomas that arose exclusively from SOC and compare it with already published data, in order to determine prognostic factors for overall and disease-free survival (DFS).

## Methods

### Study design and patient selection

This is a cohort study including 214 secondary peripheral chondrosarcomas originating from solitary osteochondromas diagnosed in a multidisciplinary setting at the *IRCCS Istituto Ortopedico Rizzoli* and retrospectively identified from 1943 to 2019. An origin from a solitary osteochondroma was confirmed clinically and radiologically by the absence of other osteochondromas.

Clinical data, including gender, age, site of the neoplasm, surgical treatment and status of surgical margins, were collected for each case from the digital or physical files. The types of treatment: complete resection, partial resection, debulking and amputation correspond, respectively, to the definition of margins of the Musculoskeletal Tumor Society as defined by Enneking et al. [8]: wide (the presence of normal tissue between tumor and margin), marginal (resection along the pseudocapsule or reactive zone around the tumor), intralesional (macroscopic or microscopic tumor at the margin) and radical (an entire anatomical compartment excised).

Follow-up data and status of patients at last follow-up were available and updated to their last visit in the digital archive.

The anatomical site of the neoplasms was established by radiology. Maximum linear dimension and cartilaginous caps of tumors were measured by CT and/or MRI or by gross examination.

**Fig. 1:**
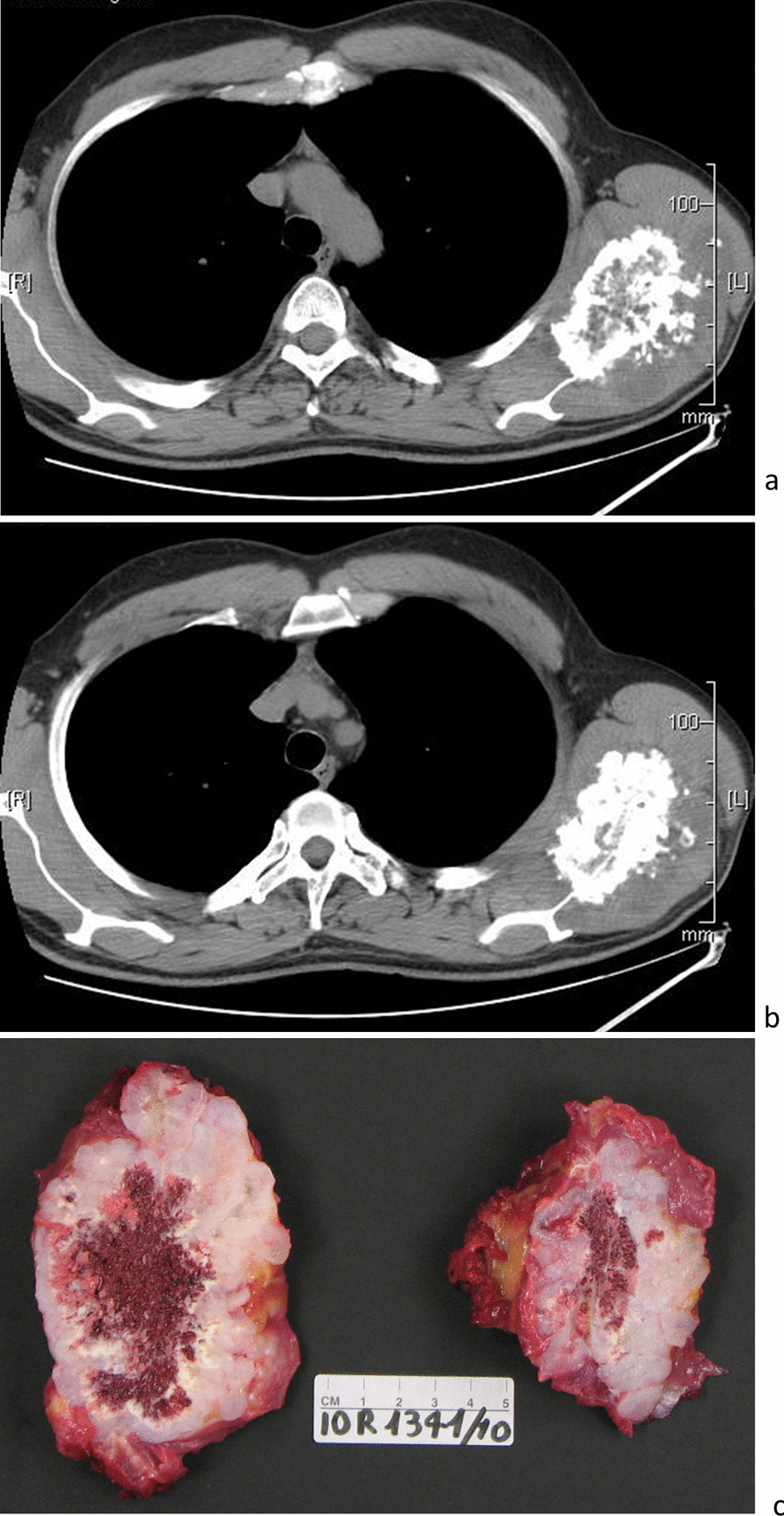
Radiological and macroscopic images: an example of PSC case of iliac wing"

Figure 1a–c show radiological and macroscopic images of a PSC.

All haematoxylin and eosin (H&E)-stained sections were reviewed by four pathologists (Marco Gambarotti, Marina Pacheco, Sofia Asioli and Alberto Righi). The histological grade was confirmed or re-assessed according to the World Health Organization (WHO) grading for central chondrosarcomas [1] without knowledge of the outcome. Ethical committee approval was obtained from the Comitato Etico di Area Vasta Emilia Centro on 22/10/2020 (Codice CE AVEC 929/2020/Oss/IOR).

### EXT1/EXT2 molecular screening

Patients with one or more relatives with a diagnosis of solitary osteochondroma were evaluated for the presence of germline heterozygous variants in *EXT1* and *EXT2* genes to exclude hereditary MO. All coding exons and flanking exon–intron junctions of *EXT1* and *EXT2* were evaluated by Sanger sequencing using the ABI PRISM 3500XL Genetic Analyzer (Thermo Fisher Scientific, Waltham, MA, USA) for the presence of point mutations. In case of negative result, both genes were evaluated by MLPA (Multiple Ligation-dependent Probe Amplification) analysis for the presence of exon or multi-exons deletion/amplification as previously described [9].

### Statistical analysis

Patients’ characteristics were reported as medians (along with interquartile range, IQR) and percentages for continuous and categorical variables, respectively. The association between categorical variables were evaluated using Fisher's exact test.

Overall survival (OS) was calculated from diagnosis to death from all causes. DFS was calculated from diagnosis to local recurrence, metastasis onset or death due to disease. OS and DFS were estimated using the Kaplan–Meier method and compared between groups using the log-rank test. Hazard ratios (HRs) were estimated using the Cox proportional hazard model. The effect of the local recurrence and metastases onset on OS was evaluated with a Cox model using time-varying variables, splitting the follow-up of patients with respect to the onset or not of one of the two outcomes.

A multivariable Cox model for OS and DFS that included all the patients was performed using multiple imputation to account for missing data in the predictors that were evaluated. Combined estimates were obtained from 25 imputed data sets. The potential nonlinear relationship of maximum tumor dimension and cartilage thickness with OS and DFS was previously evaluated with the Cox model using the restricted cubic splines transformations. In addition, since the two variables were strongly correlated (Pearson's rho = 0.694), their effects were not adjusted to each other in multivariable analysis.

## Results

A total of 214 patients with secondary chondrosarcoma arising from SOC were identified in our database during the study period. Table 1 summarizes the patients and tumors´ features. The median age at diagnosis was 38 (IQR 27–49). There were 142 (66.4%) male and 72 (33.6%) females (M: F = 2:1).

**Table 1:** Patients and tumors characteristics

Factor	Level	Value (%)
Number of cases		214
Sex	F	72 (33.6%)
	M	142 (66.4%)
Median age at diagnosis (IQR)^a^		38 (27–49)
Histological grade	1	112 (52.4%)
	2	98 (45.7%)
	3	4 (1.9%)
Axial	No	78 (36.4%)
	Yes	136 (63.6%)
Median cartilage cap thickness (cm) (IQR)^b^		3 (2–5)
Median maximum tumor dimension (cm) (IQR)^c^		10 (7–14.5)
Surgical treatment	Amputation	12 (5.6%)
	Complete resection	80 (37.4%)
	Debulking	53 (24.8%)
	Partial resection	54 (25.2%)
	Unknown	15 (7.0%)
Local recurrence/metastasis	No	165 (77.1%)
	Yes	49 (22.9%)
Outcome at last follow-up	Alive	177 (82.7%)
	Dead of disease	24 (11.2%)
	Dead of other/unknown causes	13 (6.1%)

F, female; M, male

^a^1 missing

^b^65 missing

^c^40 missing

EXT1 and EXT2 mutational analyses performed in 3 patients with a positive family history for solitary osteochondroma did not detect any pathogenic variant, so excluding hereditary MO.

**Fig. 2:**
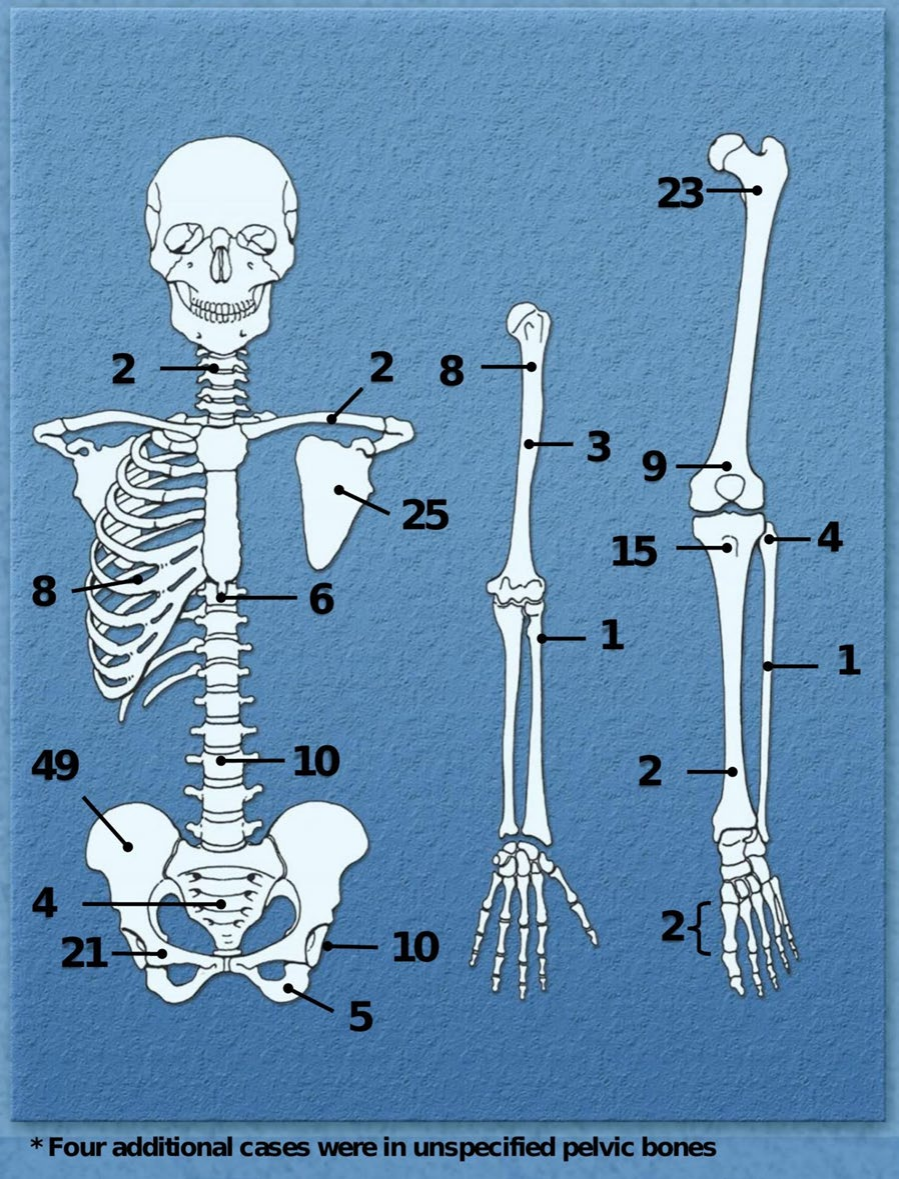
Anatomical distribution of secondary peripheral chondrosarcomas

Anatomic distribution of all cases is shown in Fig. 2. Axial tumors (136; 63.6%) outnumbered appendicular-based tumors (78; 36.4%). The most prevailing primary site was the pelvic girdle (79 cases, 36.9%), distributed as follows: iliac wing, 49 cases; pubic bone, 21 cases; ischium, 5 cases and pelvis not specified, 4 cases. Other relatively common sites in order of frequency were femur (32; 14.9%), scapula (25; 11.7%), tibia (17; 7.9%) and humerus (11; 5.1%).

As assessed on the histological sections from the surgical specimens, most of the primary tumors were grade 1 (112; 52.4%), followed by grade 2 (98; 45.7%). A minority were grade 3 (4; 1.9%).

The thickness of the cartilaginous cap was reported in 149 tumors. The median thickness was 3 cm (IQR 2.0 to 5.0; range 1.0–14.0). The median maximum tumor dimension was 10 cm (IQR 7–14.5; range 2.5–44).

The primary surgical treatment was as follows: complete resection (80; 37.4%), partial resection (54; 25.2%), debulking (53; 24.8%) and amputation (12; 5.6%). The primary surgical procedure was not reported in 12 patients (5.6%) and three patients (1.4%) had inoperable tumors at diagnosis. Resection margins were wide in complete resections; radical, in amputations; marginal in partial resections and intralesional in debulking procedures.

Follow up data was available for all patients. The median follow-up time was 11.1 years (IQR 5.3–19.7). At the time of the latest follow-up, 177 patients (82.7%) were alive, 24 (11.2%) died of disease and 13 (6.1%) died of unknown or non-related causes.

One hundred and forty-nine patients (69.6%) were disease-free after primary surgery during the follow-up period; 24 patients (11.2%) had no evidence of disease following treatment for local recurrences or metastases, and four patients (1.9%) were alive with disease at latest follow-up.

Overall, the 5-year and ten-year OS were 92.3% and 84.9%, respectively. Besides age (< 50), which is, generally speaking, a strong factor of increased overall survival, high histologic grade was the only other factor associated with worse five-year and 10-year OS (log-rank *p* = 0.0005, HR = 3.74; 95%CI 1.69–8.26) (Table 2). Of those patients who died of disease**,** 2/24 (8.3%) had grade 1 tumors and 22/24 (91.7%), had grades 2 and 3 tumors.

**Table 2:** Kaplan-Maier estimates of OS by patient characteristics

Factor	Level	Kaplan–Maier estimates (95%CI)		Log-rank *p* value	HR (95%CI)
		5-year	10-year		
Gender	F (ref)	93.8 (84.2–97.6)	87.0 (74.2–93.7)	0.583	1
	M	91.5 (84.7–95.3)	83.7 (74.8–89.6)		1.21 (0.61–2.42)
Age	< 30 (ref)	93.2 (82.9–97.4)	88.8 (76.4–94.9)	0.0262	1
	30–50	94.2 (86.5–97.5)	88.1 (78.2–93.7)		1.53 (0.65–3.62)
	> 50	86.6 (70.7–94.2)	70.4 (49.7–83.9)		3.08 (1.27–7.46)
Histological grade	1 (ref)	97.0 (91.0–99.0)	95.6 (88.6–98.4)	0.0005	1
	≥ 2	86.9 (77.6–92.6)	73.5 (61.7–82.1)		3.74 (1.69–8.26)
Axial	No (ref)	97.0 (88.3–99.2)	89.9 (82.9–94.1)	0.226	1
	Yes	90.0 (77.2–95.8)	82.3 (73.5–88.4)		1.41 (0.68–2.91)
Type of surgery	Amputation	87.5 (38.7–98.1)	87.5 (38.7–98.1)	0.3616	1.08 (0.14–8.57)
	Complete resection (ref)	95.8 (87.5–98.6)	90.2 (79.2–95.5)		1
	Debulking	91.5 (78.8–96.7)	82.4 (66.1–91.4)		1.56 (0.60–4.06)
	Partial resection	91.2 (78.3–96.6)	82.2 (65.8–91.3)		2.10 (0.90–4.89)
Cartilage cap	< 3 cm (ref)	96.9 (88.1–99.2)	92.4 (80.7–97.1)	0.2524	1
	≥ 3 cm	93.7 (83.9–97.6)	86.7 (73.6–93.6)		1.88 (0.63–5.61)
Max. tumor dimension	< 15 (ref)	93.7 (87.3–97.0)	88.8 (80.5–93.7)	0.3423	1
	≥ 15	94.4 (79.5–98.6)	83.7 (64.9–93)		1.52 (0.64–3.61)

**Fig. 3:**
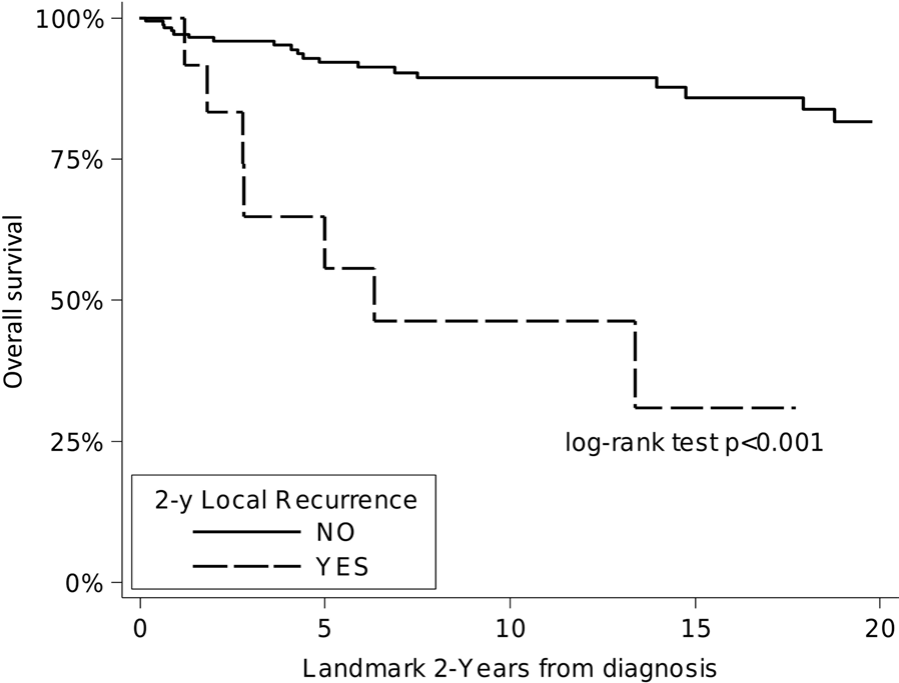
Time-varying variables analysis of OS by local recurrence within 2-year from diagnosis

According to the analysis performed using time-varying variables, the risk of death of patients seems to increase largely after local recurrence (HR = 7.40; 95% CI = 3.65–15.01) rather than after distant progression (HR = 2.76; 95%CI 0.60–12.63) (Fig. 3).

There were no significant differences in OS between patients with axial or limb-based tumors (*p* = 0.226), type of primary surgical treatment (*p* = 0.3616), maximum tumor dimension (*p* = 0.3423) and thickness of cap (*p* = 0.2524).

The five-year and ten-year DFS were 80.7% and 73.7%, respectively. During the follow-up period, 49 (22.9%) patients had events of disease progression after the primary surgical treatment. Overall, the rate of local recurrences was 17.3% and the rate of metastases was 5.1%. The cohort of patients with disease progression included 12/112 (10.7%) patients with grade 1 tumors; 35/98 (35.7%) with grade 2 and 2/4 (50%) with grade 3 tumors.

The median maximum dimension of the tumors that progressed was 11 cm (IQR 9.0–17.0; range 1.0–44.0). The median thickness of the cartilage cap was 5 cm (IQR 3.25–8.25; range 1.8 cm to 12.0). The type of primary surgical treatment was available in 41/49 patients that had disease progression and included 15/54 (27.8%) patients that underwent partial resections, 15/53 (28.3%) patients that underwent debulking; 7/80 (8.75%) patients with complete resections and 4/12 (33.3%) patients that were amputated.

The histologic grade of the recurrent or metastatic tumors was available in 39 cases. Most progressions were the same grade as the primary tumor, only four patients (10%) had tumors progressing to a higher grade.

Information about the site of progression was available in 45/49 patients; of these, 34 experienced local recurrences (75.6%); eight, distant metastases (17.7%) and three had both (6.7%). Information regarding the number of progression events was available in 46 patients. Overall, 27 (58.7%) had single events and 19 (41.3%), two or more. The median time from primary treatment to first event of progression was 32.5 months (IQR 12–59; range 1–207), although multiple events of progression occurred up to 296 months after primary surgical treatment.

Of the 34 patients with local recurrences, 13 patients (38.2%) experienced two or more events with a median of 2 local recurrences per patient (IQR = 2–3; range = 2–5). Local recurrences were re-excised and at latest follow up, control was achieved in 18/34 (52.9%) patients; 11/34 (32.4%) patients died due to the effect of local progressions on surrounding organs and structures; one (2.9%), was alive with disease after re-excision and four (11.8%) died of unknown causes.

Metastasis were to lung (6), regional lymph nodes (2), kidney (1), pelvic soft tissues (1) and abdominal wall (1).

**Fig. 4:**
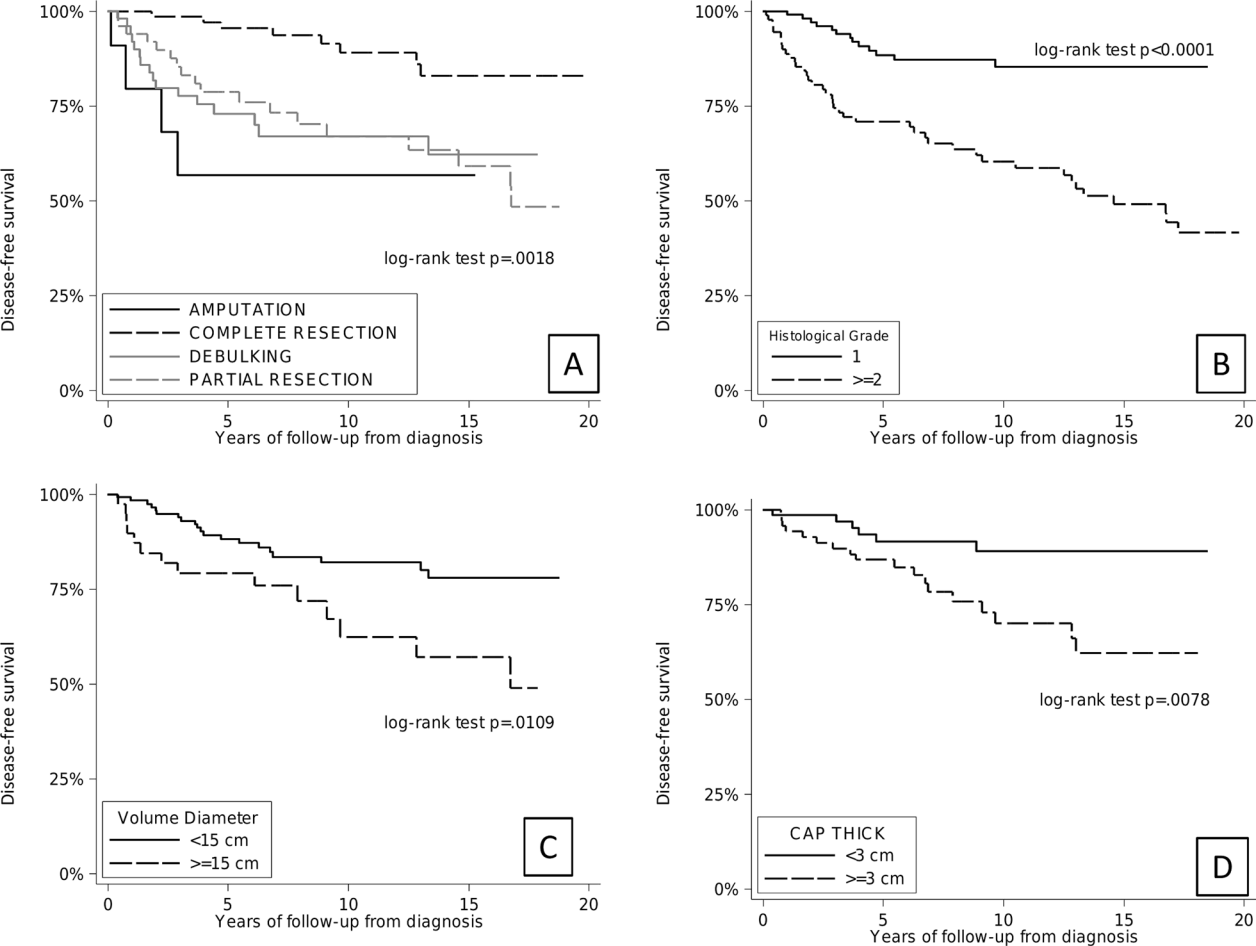
Kaplan–Meier curves comparing: **a** disease-free survival by type of primary surgical resection (*p* = .0018); **b** disease-free survival split by whether the histological grade was G1 or G2 or greater (*p* = .0001); **c** disease-free survival split by whether the maximum tumor dimension was < or > 15 cm (*p* = .0109); **d** disease-free survival split by whether the cartilaginous cap was < or > 3 cm (*p* = .0078)

Univariate associations of characteristics of patients with DFS are reported in Fig. 4a–d).

High histological grade (HR = 3.75; 95% CI = 1.69–8.34; *p* = 0.001) and surgical debulking (HR = 3.71; 95% CI = 1.57–8.79; *p* = 0.003) were the two variables associated with a significatively worse DFS that confirm significance also in multivariable analysis (Table 3). Instead the size of the tumor and the thickness of the cartilaginous caps did not maintain significant association with DFS in multivariate analysis.

**Table 3:** Multivariable analysis on DFS (recurrence/metastasis free survival)

Variables	HR^a^	95%CI	*P* value
Males	0.85	0.48–1.53	0.589
Age, per 1-year increase	1.00	0.98–1.02	0.990
Histological grade ≥ 2	3.75	1.69–8.34	0.001
Axial	1.03	0.56–1.91	0.925
Amputation versus complete resection	1.27	0.36–4.52	0.713
Debulking versus complete resection	3.71	1.57–8.79	0.003
Partial resection versus complete resection	1.37	0.56–3.35	0.490
Cartilage cap thickness (cm), per 1-cm increase	1.10	0.93–1.28	0.258
Max tumor dimension, per 1-cm increase	1.02	0.95–1.09	0.566

^a^HR unadjusted to each other in multivariable analyses

## Discussion

This retrospective, single institutional series of secondary peripheral chondrosarcomas arising from solitary osteochondromas constitutes the experience of Rizzoli during a study period of almost 80 years.

In the current study we found a rate of local recurrence of 17.3%, which is similar to rates previously reported by some authors [4, 5], although considerably lower than recurrence rates shown by Tsuda et al. (29%) [7] and Garrison et al*.* (52%) [6] The inclusion of patients with MO in previous reports might, at least in part, account for the higher rates of worse local outcomes, as it has been shown that secondary peripheral chondrosarcomas in the context of multiple osteochondromas have higher rates of local recurrences and significantly worse local recurrence-free survivals [6, 7]. Our rate of metastases was 5.1% which confirms the low-grade behavior of peripheral secondary chondrosarcomas, a neoplasm that consistently has displayed a low metastatic rate in previous series.

In general, 5 and 10-year OS in our study was 92.3% and 84.9%, respectively. Our results fall within the range of previously published 10-year DSS for peripheral secondary chondrosarcomas, which vary between 70 and 90% [4, 7].

Our statistical analysis shows that high histologic grade negatively affects the OS in patients with secondary peripheral chondrosarcomas. The mortality rate from disease was strikingly lower for grade 1 tumors (1.8%) than for grade 2 and 3 tumors (21.6%). The 5 and 10-year OS are 97% and 95% for patients with grade 1 tumors and 86.9% and 73.5% for patients with grade 2 and 3 tumors, respectively. In multivariate analysis, grade is also one of the factors independently associated with worse DFS. The published data on the effect of histological grade on the prognosis of peripheral secondary chondrosarcoma is scanty, conflicting and mostly derived from observational studies. The conclusions made range from histological grade being a subordinate factor to the adequacy of margins on the rate of local recurrences [4], to play an indefinite role in prognosis due to low number of cases [5, 6] to the recognition of high histological grade as a negative prognostic factor for local recurrence free survival but not for DSS [7]. Based on our results, the impact of histological grade on the survival of patients with peripheral secondary chondrosarcoma is evident and highlights the importance of accurately grading these neoplasms. The assessment of histological grade for cartilaginous tumors bears difficulties and it is well-known to vary considerably among pathologists [10]. Most specifically, the distinction between enchondroma and grade I chondrosarcoma and the differentiation between grade I and grade II chondrosarcomas [11]. Additionally, in the context of cartilaginous neoplasms of the bone surface, the relevance of histological grade on the survival of secondary peripheral chondrosarcoma renders pivotal the distinction of it from a periosteal chondrosarcoma, as the outcome of the latest is not affected by high histologic grade, based on published data [12–14] and our personal observations.

Patients whose tumors were treated by complete resection with wide margins had the highest DFS in univariate analysis. In multivariable analysis, the two factors that independently worsened the DFS were high histologic grade and intralesional margins. We concur with authors of the previous series on secondary peripheral chondrosarcomas that the adequate primary surgical approach must be wide resection irrespective of the location of the tumor, when feasible. Our results indicate that local recurrences clearly have a negative influence on OS and that patients with secondary peripheral chondrosarcomas die of consequences of unresectable local tumor recurrences, thus a successful primary surgical treatment is critical to maintain local recurrences low which in turn would improve long term survival. We do not see a trend of local recurrences influencing the rate of metastatic disease.

From our results, the first event of progression of secondary peripheral chondrosarcoma occurs at a median time of 32.5 months and, half of the cases that recur, do so at an interval between one and 6 years after the primary surgical treatment. Of those patients that progress, 41.3% experience two or more events of local or distant recurrences and some remain at risk for as long as 24 years after initial treatment. As it is the case for central conventional chondrosarcomas [15–17], the protracted nature of secondary peripheral chondrosarcoma renders long-term follow up necessary.

The large number of cases, their evaluation and revision by four pathologists and the study period of almost 80 years represent factors of strength of the study. Indeed, some limitations of the current study must be acknowledged.

First, the retrospective design of this series that spreads over seven decades, introduces potential differences in diagnostic methods and treatment. However, given the rarity of this tumor, a prospective study would be difficult. Another limitation is missing data. Specifically, variables such as cartilage cap thickness and maximum tumor dimension, were not collected in the first place, thus in many cases such information was unretrievable. Finally, an inherent limitation of retrospective series of cases is that an adequate control group is not possible and cofounding variables are difficult to measure, so it is not feasible to make causal statement.

## Conclusions

Our large series of 214 secondary peripheral chondrosarcomas arising in solitary osteochondromas highlights that the best choice of treatment is wide surgical excision, when possible. Location per se is not a factor that affects prognosis. An accurate assessment of the histological grade is of outmost importance, as it is correlated with tumor aggressiveness and survival. A Long term follow up is necessary after primary treatment for this rare variant of chondrosarcoma.

## Data Availability

The datasets used and/or analyzed during the current study are available from the corresponding author on reasonable request.

## Abbreviations

DFS: Disease-free survival
DSS: Disease-specific survival
H&E: Haematoxylin and eosin
HRs: Hazard ratios
IQR: Interquartile range
MLPA: Multiple ligation-dependent probe amplification
MO: Hereditary multiple osteochondromas
OS: Overall survival
SOC: Malignant transformation of solitary osteochondromas
SPCs: Secondary peripheral chondrosarcomas
WHO: World Health Organization
